# Genetic Analysis of Human Norovirus Strains in Japan in 2016–2017

**DOI:** 10.3389/fmicb.2018.00001

**Published:** 2018-01-18

**Authors:** Koo Nagasawa, Yuki Matsushima, Takumi Motoya, Fuminori Mizukoshi, Yo Ueki, Naomi Sakon, Koichi Murakami, Tomomi Shimizu, Nobuhiko Okabe, Noriko Nagata, Komei Shirabe, Hiroto Shinomiya, Wataru Suzuki, Makoto Kuroda, Tsuyoshi Sekizuka, Yoshiyuki Suzuki, Akihide Ryo, Kiyotaka Fujita, Kazunori Oishi, Kazuhiko Katayama, Hirokazu Kimura

**Affiliations:** ^1^Infectious Disease Surveillance Center, National Institute of Infectious Diseases, Musashimurayama, Japan; ^2^Department of Pediatrics, Graduate School of Medicine, Chiba University, Chiba, Japan; ^3^Division of Virology, Kawasaki City Institute for Public Health, Kawasaki, Japan; ^4^Division of Virology, Ibaraki Prefectural Institute of Public Health, Mito, Japan; ^5^Department of Microbiology, Tochigi Prefectural Institute of Public Health and Environmental Science, Utsunomiya, Japan; ^6^Division of Virology, Department of Microbiology, Miyagi Prefectural Institute of Public Health and Environment, Sendai, Japan; ^7^Department of Microbiology, Osaka Institute of Public Health, Osaka, Japan; ^8^Yamaguchi Prefectural Institute of Public Health and Environment, Yamaguchi, Japan; ^9^Department of Microbiology, Ehime Prefectural Institute of Public Health and Environmental Science, Matsuyama, Japan; ^10^Eiken Chemical Co., Ltd., Biochemical Research Laboratory I Department-I, Shimotsuga, Japan; ^11^Pathogen Genomics Center, National Institute of Infectious Diseases, Shinjuku, Japan; ^12^Division of Biological Science, Department of Information and Basic Science, Graduate School of Natural Sciences, Nagoya City University, Nagoya, Japan; ^13^Department of Microbiology, Yokohama City University School of Medicine, Yokohama, Japan; ^14^School of Medical Technology, Faculty of Health Science, Gunma Paz University, Takasaki, Japan; ^15^Laboratory of Viral Infection I, Kitasato Institute for Life Sciences, Graduate School of Infection Control Sciences, Kitasato University, Minato, Japan

**Keywords:** norovirus, capsid, RNA-dependent RNA polymerase, phylogeny, molecular evolution, epitope mapping

## Abstract

In the 2016/2017 winter season in Japan, HuNoV GII.P16-GII.2 strains (2016 strains) emerged and caused large outbreaks of acute gastroenteritis. To better understand the outbreaks, we examined the molecular evolution of the *VP1* gene and *RdRp* region in 2016 strains from patients by studying their time-scale evolutionary phylogeny, positive/negative selection, conformational epitopes, and phylodynamics. The time-scale phylogeny suggested that the common ancestors of the 2016 strains *VP1* gene and *RdRp* region diverged in 2006 and 1999, respectively, and that the 2016 strain was the progeny of a pre-2016 GII.2. The evolutionary rates of the *VP1* gene and *RdRp* region were around 10^-3^ substitutions/site/year. Amino acid substitutions (position 341) in an epitope in the P2 domain of 2016 strains were not found in pre-2016 GII.2 strains. Bayesian skyline plot analyses showed that the effective population size of the *VP1* gene in GII.2 strains was almost constant for those 50 years, although the number of patients with NoV GII.2 increased in 2016. The 2016 strain may be involved in future outbreaks in Japan and elsewhere.

## Introduction

Human norovirus (HuNoV) is a common cause of acute gastroenteritis in humans (Matson, 2004). HuNoV is classified into two genogroups (GI and GII), including many genotypes (Vinjé, 2015). Of GII’s confirmed 22 genotypes (GII.1-GII.22) (Vinjé, 2015), GII.4 was a major cause of pandemics in 2006–2012 (Siebenga et al., 2007; Eden et al., 2014). Furthermore, other some genotypes, including GII.2, GII.3, GII.6, and GII.17, were detected worldwide, including Japan (Thongprachum et al., 2017).

During the 2016–2017 winter season in Japan, GII.P16-GII.2 strains (2016 strains) suddenly appeared and caused large outbreaks of gastroenteritis in Japanese children (Thongprachum et al., 2017). The Japanese national surveillance system reported in Infectious Diseases Weekly Report (IDWR) that the outbreaks of acute gastroenteritis in children in this season were the second largest number of patients in the past 11 seasons [National Institute of Infectious Diseases, Japan, Infectious Gastroenteritis (cited 2017 February 16, in Japanese)^1^]. Similar findings were reported from Germany, France, China, and United States (Bidalot et al., 2017; Lu et al., 2017; Niendorf et al., 2017; Tohma et al., 2017).

To better understand the genetics of the 2016 strains, we completed a detailed analysis of the full length of the RNA-dependent RNA polymerase (*RdRp*) region and capsid (*VP1*) gene of the GII.P16-GII.2 strains detected in Japan during 2016–2017 winter season.

## Materials and Methods

### Samples and Patients

In the present study, we analyzed 19 strains of the 2016 strains. Samples were collected from patients (children and adults) with acute gastroenteritis (9.2 years ± 12.4, mean ± standard deviation) from October to December in 2016. The samples were obtained from clinics or hospitals, such as the sentinel surveillance medical institutions in Ibaraki prefecture and Kawasaki city in Japan. Written informed consent was obtained from the patients or their guardian for the donation of samples. The study protocols were approved by National Institute of Infectious Diseases for Public Health Ethics Committees (No. 576).

### RNA Extraction, Reverse Transcription Polymerase Chain Reaction, and Sequencing

Viral RNA was extracted from samples with a QIAamp Viral RNA Mini kit (Qiagen). Reverse transcription–polymerase chain reaction (RT-PCR) was performed using a PrimeScript II High Fidelity One Step RT-PCR Kit (TaKaRa). Using primer walking methods, we analyzed the complete *RdRp* region and *VP1* gene. The primers were designed by the PrimaClade server (**Supplementary Table S1**) (Gadberry et al., 2005). After the PCR products were purified using a MinElute PCR Purification Kit (Qiagen), they were sequenced using a BigDye Terminator v3.1 Cycle Sequencing kit (Applied Biosystems). We used the Norovirus Genotyping Tool version 1.0 to genotype the present virus (Kroneman et al., 2011). The accession numbers of these strains are shown in **Supplementary Table S2**.

### Phylogenetic Analyses and Estimating the Evolutionary Rate by a Bayesian Markov Chain Monte Carlo (MCMC) Method

To examine the evolution of the strains, we collected complete sequences of the *VP1* gene and *RdRp* region of HuNoV from GenBank and constructed phylogenetic trees of the genes by the MCMC method. The full-length *VP1* gene sequences of the HuNoV GII.2 strains with the detection year were collected in December 2016. A total of 186 strains were obtained, including our present strains. Furthermore, the full-length *RdRp* region sequences of the HuNoV GII.P16 strains and HuNoV GII.2 strains with the detection year were also collected in December 2016. A total of 107 strains were obtained, including the present strains. At that time, new GII.2 variants’ sequence data detected from other areas were not disclosed in GenBank. Thus, the evolutionary analyses were performed on our strains alone. The best substitution models were selected using the BIC method by MEGA6.0 (Tamura et al., 2013). Appropriate clock and tree models were determined by the pass sampling method using the BEAST2 packages (Bouckaert et al., 2014). To generate the posterior set of trees, we used BEAST v2.4.5 (Bouckaert et al., 2014) (**Supplementary Table S3**). After the first 10% of the chain was omitted, effective sample sizes greater than 200 were accepted, as described in the manual. Maximum clade credibility trees were constructed using Tree Annotator v2.4.5, and the MCC phylogenetic trees were visualized using FigTree v1.4.0. Moreover, we estimated the evolutionary rates of the *VP1* gene and *RdRp* region using BEAST v2.4.5. Appropriate models were selected as described above.

### Calculation of Pairwise Distances of the *VP1* Gene and *RdRp* Region in HuNoV Strains

To evaluate the genetic divergence of the *VP1* gene and *RdRp* region, the pairwise distances were calculated using MEGA 6.0, as described (Tamura et al., 2013). In addition, to recognize genetic diversity among the GII.P16-GII.2 strains, we constructed phylogeny trees of the *VP1* gene and *RdRp* region using neighbor-joining trees. In these phylogenic trees, we added six strains detected in other countries in the 2016/2017 winter season as reference strains (*VP1*: KY421044, KY485115, KY485122, *VP1,* and *RdRp*: KY771081, KY865306, KY865307).

### Construction of the Capsid Protein Structural Models and Prediction of the B-Cell Conformational Epitopes in the Capsid Protein

To examine the relationship between amino acid substitutions and B-cell epitopes, we constructed structural models of the capsid protein and their predicted B-cell conformational epitopes. We made two models with the strains Hu/GII/JP/2010/GII.2/Ehime43 (one of the strains with highest identity score to the 2016 strains in the past GII.2 strains) and Hu/GII/JP/2016/GII.P16-GII.2/Kawasaki129 (2016 strain). The homology modeling was based on the crystal structures of the 1IHM and 4RPB (Protein Data Bank accession numbers). After aligning these data using MAFFTash (Katoh et al., 2002), the models were constructed using MODELER v9.15 (Webb and Sali, 2014) and adjusted using Swiss PDB Viewer v4.1 (Guex and Peitsch, 1997). Then, they were confirmed by Ramachandran plot analysis using the RAMPAGE server (Lovell et al., 2003), and the residues of putative B-cell epitopes and amino acid substitutions were mapped on the predicted structure using Chimera v1.10.2 (Pettersen et al., 2004).

Next, we predicted B-cell conformational epitopes in the capsid protein in the structural models by DiscoTope2.0 (Kringelum et al., 2012), BEPro (Sweredoski and Baldi, 2008), EPCES (Liang et al., 2007), and EPSVR (Liang et al., 2010). We used these tools with cut-offs of -3.7 (DiscoTope2.0), 1.3 (BEPro), and 70 (EPCES, EPSVR). We accepted sites as B-cell conformational epitopes when they were inferred by three or more methods. Amino acid substitutions of the strain Hu/GII/JP/2016/GII.P16-GII.2/Kawasaki129 that corresponded to the strain Hu/GII/JP/2010/GII.2/Ehime43 and predicted epitopes were mapped onto the model. Moreover, to estimate whether the amino acid substitutions in the predicted epitopes were characteristic for the 2016 strains, we examined the number of strains with these amino acid substitutions among all present GII.2 strains (186 strains), compared to Snow mountain strain.

### Selective Pressure Analysis

To estimate sites under positive or negative selection in the capsid protein of the 2016 strains, we calculated the rates of non-synonymous (dN) and synonymous (dS) substitutions at every codon position with the Datamonkey package (Delport et al., 2010). In the present study, we analyzed only the 2016 strains. We used the fixed effects likelihood (FEL) method, the internal fixed effects likelihood (IFEL), the single likelihood ancestor (SLAC), and the random effects likelihood (REL) methods.

### Bayesian Skyline Plot Analyses

To examine the changes in the effective population size of the HuNoV GII.2 strains, Bayesian skyline plot (BSP) analyses were performed using all collected strains (*VP1*: 186 strains; *RdRp*: 88 strains), including 100% identical sequences by BEAST v2.4.5, as reported (Drummond et al., 2005; Bouckaert et al., 2014). The appropriate models for these analyses were selected as described above. The detailed conditions of each analysis are shown in **Supplementary Table S4**.

## Results

### Phylogenetic and Evolutionary Rate Analyses of the *VP1* Gene in HuNoV GII.2

**FIGURE 1:**
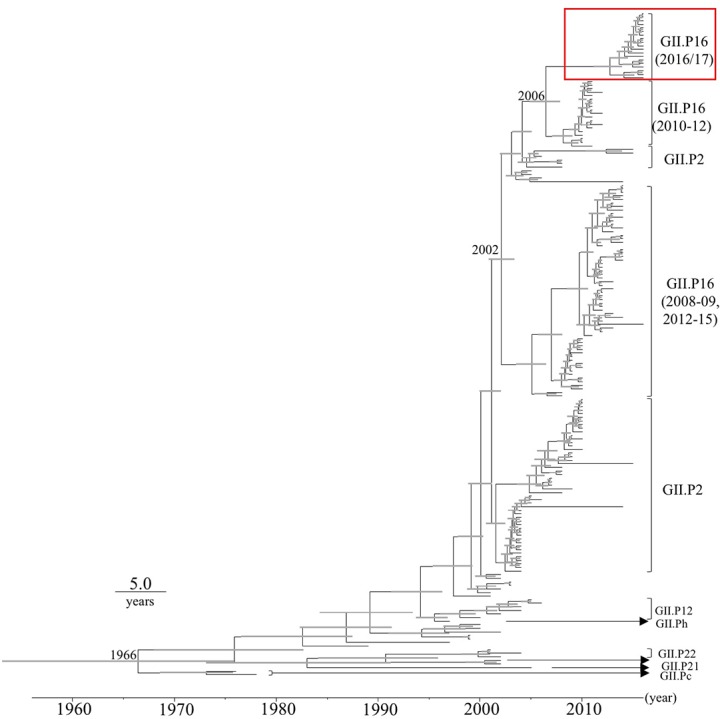
Phylogenetic tree for the *VP1* gene in the HuNoV GII strains was constructed using the Bayesian Markov chain Monte Carlo method. The scale bar represents time (years). Gray bars indicate the 95% highest probability density (HPD) for the branched year. The genotypes of the *RdRp* region of the strains are shown with the *RdRp* region sequences. A red square shows 2016 strains.

Time-scale evolutional phylogenetic trees were constructed by an MCMC method, based on the full-length nucleotide sequences of *VP1* (**Figure 1**). To perform this analysis, we used 19 2016 strains that were obtained by December 2016, and 167 reference strains from GenBank. The MCC tree of the *VP1* genes shows that the *VP1* gene was divided into many clusters. Interestingly, the *VP1* sequences clustered according to their *RdRp* sequence. The most recent common ancestor of the GII.2 strains was estimated to have circulated in 1966 [95% highest posterior density (HPD) interval, 1953–1975]. Furthermore, the most recent common ancestor of the GII.P16-GII.2 strains was in 2002 (95% HPD interval, 2000–2003). Subsequently, the common ancestors of the GII.P16-GII.2 cluster, detected in 2010–2012, and the 2016 strains diverged in 2006 (95% HPD interval, 2004–2007). The evolutionary rate of the *VP1* gene in HuNoV Gll.2 strains was estimated as 3.26 × 10^-3^ substitutions/site/year (95% HPD interval, 2.74–3.78 × 10^-3^ substitutions/site/year).

### Phylogenetic and Evolutionary Rate Analyses of the *RdRp* Region in HuNoV

**FIGURE 2:**
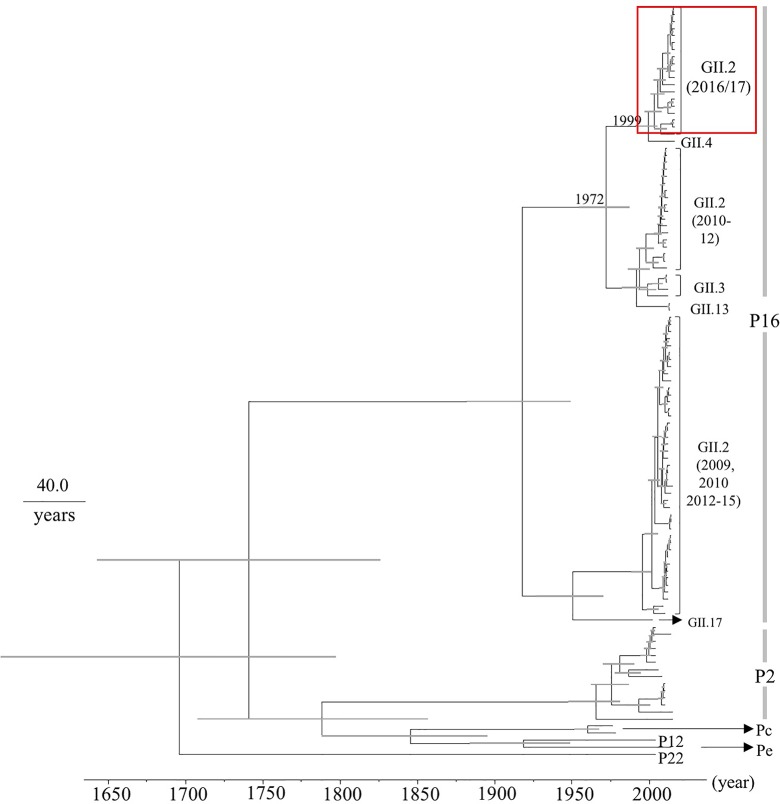
Phylogenetic tree for the *RdRp* region in the HuNoV strains was constructed using the Bayesian Markov chain Monte Carlo method. The scale bar represents time (years). Gray bars indicate the 95% HPD for the branched year. The genotypes of the *VP1* gene of the strains are shown with the *VP1* gene sequences. A red square shows 2016 strains.

Next, we determined the time-scale of the evolutionary phylogenetic tree of the various genotypes of *RdRp* region, based on their full-length nucleotide sequences (**Figure 2**). We used 19 2016 strains, including 100% identical sequences. The common ancestors of the 2016 strains, GII.P16-GII.4, GII.P16-GII.3, GII.P16-GII.13, and GII.P16-GII.2 strains detected in 2010–2012 diverged in 1972 (95% HPD interval, 1954–1987), and the common ancestors of the 2016 strains and GII.P16-GII.4 strains diverged in 1999 (95% HPD interval, 1991–2005). The evolutionary rate of the *RdRp* region in HuNoV Gll.P16 strains was estimated as 2.03 × 10^-3^ substitutions/site/year (95% HPD interval, 1.36–2.75 × 10^-3^ substitutions/site/year). These results suggest that the *RdRp* region and the *VP1* gene may have evolved independently.

### Pairwise Distances of the *VP1* Gene and *RdRp* Region Sequences

**FIGURE 3:**
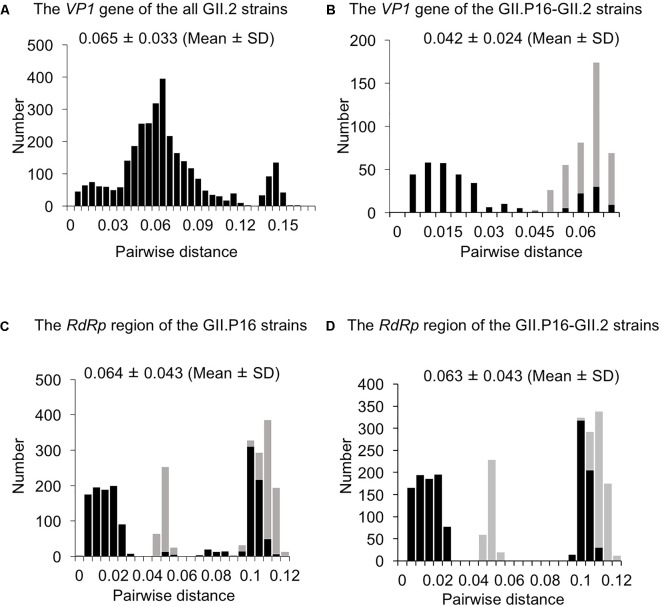
Distribution of pairwise distances between HuNoV strains for **(A)** the *VP1* gene of the all GII.2 strains, **(B)** the *VP1* gene of the GII.P16-GII.2 strains, **(C)** the *RdRp* region of the GII.P16 strains, and **(D)** the *RdRp* region of the GII.P16-GII.2 strains, based on the nucleotide sequences. The pairwise distances between the 2016 strains and the other strains are shown by gray in **(B–D)**.

To evaluate the genetic divergence between the 2016 strains and the other GII.2 strains, we calculated pairwise distances as the genetic distances among the present strains (**Figures 3A–D** and **Supplementary Figures S1a,b**). The pairwise distances values of the *VP1* gene among all GII.2 strains were 0.065 ± 0.033 (mean ± SD), and the histogram was bimodal (**Figure 3A**). Furthermore, the pairwise distances of the *VP1* genes in the GII.P16–GII.2 strains were 0.042 ± 0.0024, and the histogram was also bimodal (**Figure 3B**). The pairwise distances between the 2016 strains and the other GII.P16–GII.2 strains were relatively long (>0.043), and those of the *RdRp* region among all GII.P16 strains were 0.064 ± 0.043 (**Figure 3C**). Moreover, the pairwise distances between the 2016 strains and the other GII.P16 strains were also relatively long (>0.041). In contrast, pairwise distances between the 2016 strains and GII.P16-GII.2 strains detected in America and China in the 2016/2017 winter season were very short (**Supplementary Figures S1a,b**). These results suggested that the 2016 strains evolved further from pre-2016 GII.P16-GII.2 strains genetically.

### Structural Models of the Capsid Protein and the Predicted B-Cell Conformational Epitopes

**FIGURE 4:**
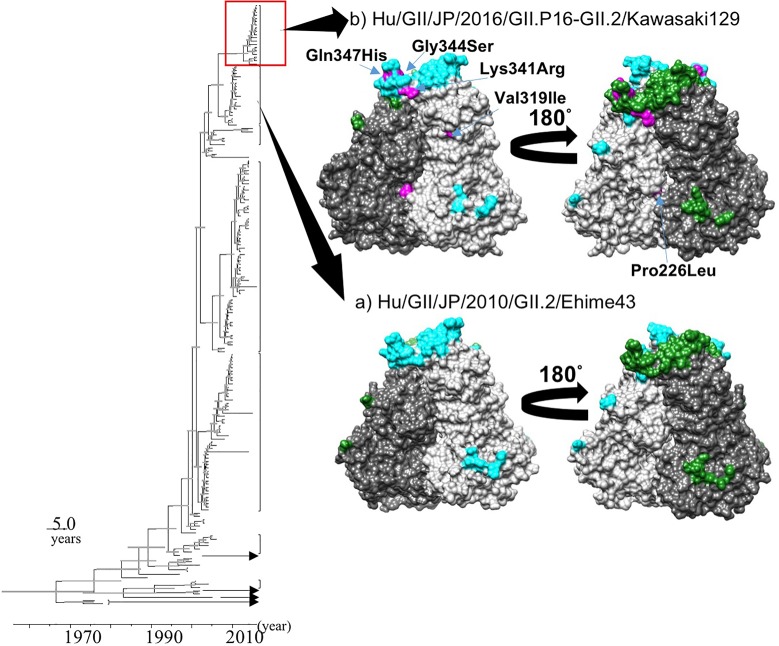
Structural models of the capsid protein in strains **(A)** Hu/GII/JP/2010/GII.2/Ehime43, and **(B)** Hu/GII/JP/2016/GII.P16-GII.2/Kawasaki129. Chains **(A)** and **(B)** are colored by gray and dim gray, respectively. The predicted conformational epitopes are shown in cyan (chain **A**) and green (chain **B**). Amino acid substitutions in the protein corresponding to the strain Hu/GII/JP/2010/GII.2/Ehime43 are indicated in magenta.

We constructed two structural models of the capsid protein (**Figures 4A,B**). Ramachandran plots showed that over 94% of the residues in each model were evaluated as geometrically favored regions. Using these models, we predicted the B-cell conformational epitopes in the capsid protein, and the predicted epitopes were mapped on the models. The prediction of the B-cell epitopes from two predicted structural models resulted in similar results (**Figure 4**). As shown in **Figure 4**, Hu/GII/JP/GII.P16-GII.2/Kawasaki129 strains had five amino acid substitutions on the surface of the structural model that corresponded to the strain Hu/GII/JP/2010/GII.2/Ehime43 (**Figure 4B**). Moreover, three amino acid substitutions (Lys341Arg, Gly344Ser, and Gln347His) were detected in the epitope on the protruding 2 (P2) domain. Among them, 14 2016 strains have an amino acid substitution at position 341 (Lys341Arg: 12 strains, Lys341Thr: two strains), but no pre-2016 GII.P16-GII.2 strains had this substitution. These results suggested that the VP1 protein of the 2016 strains has multiple amino acid substitutions in these epitopes, compared with the pre-2016 GII.P16-GII.2 strains.

### Positive and Negative Selection Site Analyses

Positive and negative selection sites in the capsid protein of the 2016 strains were inferred using FEL, IFEL, SLAC, and REL models. As a result, no positive selection sites and three negative selection sites (aa 234, 429, and 439) were estimated by FEL, but no positive or negative selection site was estimated by the SLAC, IFEL, and REL methods. These results suggested that the sequence variation of the capsid gene of 2016 strains was influenced by natural evolution rather than by selective pressure from the host immune system.

### Bayesian Skyline Plot Analyses

**FIGURE 5:**
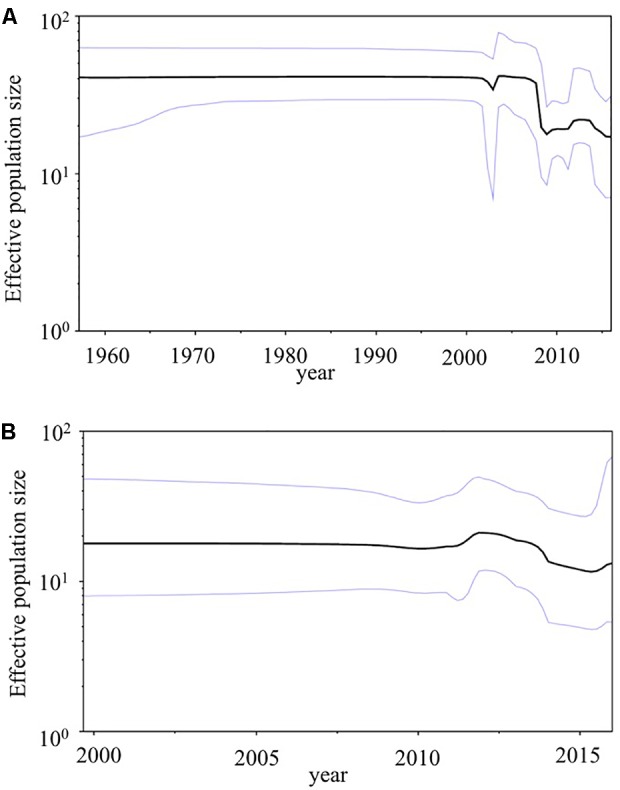
Phylodynamics of the HuNoV. **(A)** The *VP1* gene in the present GII.2 strains, and **(B)** the *RdRp* region in the present GII.P16 strains using Bayesian skyline plot analyses. The black lines indicate median effective population sizes, and the blue lines indicate 95% highest probability density.

We used BSP analyses to estimate the fluctuations of the effective population sizes for the HuNoV (**Figure 5**). The effective population size of the *VP1* gene in the HuNoV GII.2 strains was nearly constant until the year 2005. After that, the population size dropped sharply (**Figure 5A**). The fluctuation of the effective population size of *RdRp* region in the GII.P16 strains was almost constant since the year 2000 (**Figure 5B**). We could not perform BSP analyses of the 2016 strains due to their small number. These results suggest that GII.2 strains have been constantly distributed to humans since 1960, although the numbers of GII.2 detected increased in 2016 with the emergence of the 2016 strains.

## Discussion

Here we describe the evolution of the *VP1* gene and *RdRp* region in the HuNoV GII.P16-GII.2 strains detected in the 2016/2017 winter season (2016 strains) that suddenly emerged in Japan. Our main findings are as follows. (1) A common ancestor of the *VP1* gene of the 2016 strains diverged from another GII.P16-GII.2 strain around 10 years ago with an evolutionary rate as described (Mizukoshi et al., 2017) (**Figure 1**). (2) A common ancestor of the *RdRp* region of the 2016 strains diverged from the *RdRp* region of the GII.P16-GII.4 rather than the previous GII.P16-GII.2 strains in 1999 with a rapid evolutionary rate (around 10^-3^ substitutions/site/year) (**Figure 2**). Some reports estimated that the GII.P16-GII.2 strains detected in 2016 were a new recombinant strain, and both the *RdRp* region and *VP1* gene sequences of our strains and the new strains were very similar (identity: about 98–99%, data not shown), although the analyzed sequence lengths were different (Bidalot et al., 2017; Lu et al., 2017). Based on these reports, the 2016 strains might be a new chimeric virus. For example, it might be a recombination of GII.P16-GII.4 and the previous GII.P16-GII.2 (2010–2012 type) viruses, although only a limited number of strains were used in the present MCC tree.

We found that the rate of the HuNoV *VP1* gene in GII.2 strains, including 2016 strains was 3.26 × 10^-3^ substitutions/site/year. However, previous studies showed different evolutionary rates (Eden et al., 2014; Kobayashi et al., 2016; Mizukoshi et al., 2017). For example, Kobayashi et al. (2016) suggested it was around 3.76 × 10^-3^ substitutions/site/year for the *VP1* gene of all HuNoV GII. Another report showed that the GII.4 variant strains (GII.4v Sydney 2012 strains, GII4v New Orleans 2009 strains, and Apeldoorn 2007 strains) had an evolutionary rate of 7.20 × 10^-3^ substitutions/site/year (Eden et al., 2014). A very recent report showed that the rate of pre-2016 GII.2 *VP1* gene was 2.987 × 10^-3^ substitutions/site/year (Mizukoshi et al., 2017). Thus, the *VP1* gene of the present 2016 strains may evolve as well as the previous GII.2 strains (Mizukoshi et al., 2017).

Next, the current structural models of the HuNoV capsid protein suggest that amino acid substitutions occurred in the 2016 strains. In particular, an amino acid substitution at position 341, which was involved in a predicted B-cell epitope in the P2 domain, was detected in almost all 2016 strains, but was not detected in pre-2016 GII.P16-GII.2 strains. The amino acid sequences of the P2 domain are reportedly closely related to the antigenicity of the HuNoV capsid protein (Lindesmith et al., 2012). In contrast, Swanstrom et al. (2014) suggested that the GII.2 strains have evolved very little in the blockade epitopes for 35 years. In addition, a very recent report suggested that the reactivity to the immunochromatography kits showed no differences between 2016 strains and pre-2016 strains (Nagasawa et al., 2018). From these reports, it is unclear whether the amino acid substitution in the epitope causes antigenic changes or not. To the best of our knowledge, no report has investigated HuNoV neutralization assay using infectious HuNoV particles, HuNoV susceptible cells, and human antibodies, such as the secreted IgA-related gut mucosal immune response. Further studies may be needed to clarify the cause of GII.2 outbreaks.

We also estimated the positive/negative selection sites in the *VP1* gene of 2016 strains. No positive selection site was found, but there were three negative selection sites by the FEL method. In general, positive selection sites may be responsible for the immune pressure leading to an escape mutation, and negative selection sites may prevent deterioration of antigenic function and structures (Domingo, 2006). Previous reports suggested that HuNoV GII capsid proteins may not receive strong host immune pressure, resulting in no or only small numbers of positive selection sites in these VP1 proteins (Kobayashi et al., 2016; Parra et al., 2017). Thus, the present results seem to be compatible with earlier reports, although the strains we analyzed were limited in number (Kobayashi et al., 2016; Parra et al., 2017).

In addition, we calculated genetic distances of the *VP1* gene and *RdRp* region. The present 2016 strains had relatively longer pairwise distance values than the previously detected GII.P16-GII.2 strains, and relatively short pairwise distance values were found among 2016 strains (**Figures 3B–D**). Thus, the 2016 strains had larger genetic divergences than the previously detected GII.P16-GII.2 strains. In contrast, GII.P16-GII.2 strains detected in 2016/2017 winter seasons in various areas were located in a same cluster of the phylogenetic trees (**Supplementary Figures S1a,b**). In the analyzed regions, the sequence identities of these strains detected in various countries, including the United States, China, France, Germany and Japan were also very high (identity: about 98–99%, data not shown). These results suggested that genetically similar GII.P16-GII.2 strains cause the prevalence of acute gastroenteritis in many counties (Nagasawa et al., 2018). Next, we examined the phylodynamics of the HuNoV GII.2 (**Figure 5**). Notably, the effective population sizes of the *VP1* gene seem to be almost constant or to decrease slightly, but the number of acute gastroenteritis patients with HuNoV GII.2 strains increased in 2016. From these results, the symptoms of the pre-2016 GII.2 strains might be less severe than the symptoms of the 2016 strains, although these analyses are limited by the small number of old strains. Moreover, the GII.P16 *RdRp* region was also constant. To test our hypothesis, further studies on the molecular epidemiology are needed.

Molecular epidemiological studies suggested that the HuNoV genotype GII.4 caused multiple pandemics of acute gastroenteritis in people of all ages. For example, Den Haag 2006b suddenly emerged and caused a pandemic worldwide in 2006 (Siebenga et al., 2007), and Sydney 2012 caused a pandemic in 2012 (Eden et al., 2014). Detailed evolutionary analyses of these strains showed that significant amino acid substitutions occurred in the P2 domain of the GII.4 virus responsible for that pandemic, compared with the previous GII.4 strains (Siebenga et al., 2007; Eden et al., 2014). In the 2016 strains, multiple amino acid substitutions were found in the P2 domain. Indeed, this 2016 strain caused large outbreaks of acute gastroenteritis in Japanese children. In addition, the 2016 strains caused outbreaks of acute gastroenteritis in many countries (Bidalot et al., 2017; Lu et al., 2017; Niendorf et al., 2017; Tohma et al., 2017). Therefore, these strains may cause further outbreaks in Japan and elsewhere.

## Author Contributions

HK and KK designed this study. KN, YM, TM, FM, WS, and TSe analyzed the data. YM, TM, FM, TSh, NO, and NN collected the samples. YU, NS, KM, KS, HS, MK, YS, AR, KF, and KO contributed analyses tools. KN, YM, KK, and HK wrote this paper. All authors approved the final manuscript as submitted and agree to be accountable for all aspects of the work.

## Conflict of Interest Statement

The authors declare that the research was conducted in the absence of any commercial or financial relationships that could be construed as a potential conflict of interest.

## Footnotes

1 http://www.nih.go.jp/niid/ja/10/2096-weeklygraph/1647-04gastro.html

## Supplementary Material

FIGURE S1 Phylogenetic trees for **(a)** the *VP1* gene and **(b)** *RdRp* region in the HuNoV GII.P16-GII.2 strains were constructed using the neighbor-joining method. The scale bars represent substitution per site. Bootstrap values of more than 70% were shown. Circles indicate strains detected in the United States in the 2016/2017 winter season, and the square indicates the strain detected in China in the 2016/2017 winter season.

TABLE S1 Primers used in this study.

TABLE S2 Strains used in this study.

TABLE S3 Conditions of Bayesian Markov chain Monte Carlo method for the phylogenic trees.

TABLE S4 Conditions of Bayesian skyline plot analyses.
