# Supplementary figures and images for: Genetic Analysis of Human Norovirus Strains in Japan in 2016–2017
Source: Front Microbiol. 2018 Jan 18;9:1. doi: 10.3389/fmicb.2018.00001 (PMC5778136; doi:10.3389/fmicb.2018.00001)

Fig. S1a

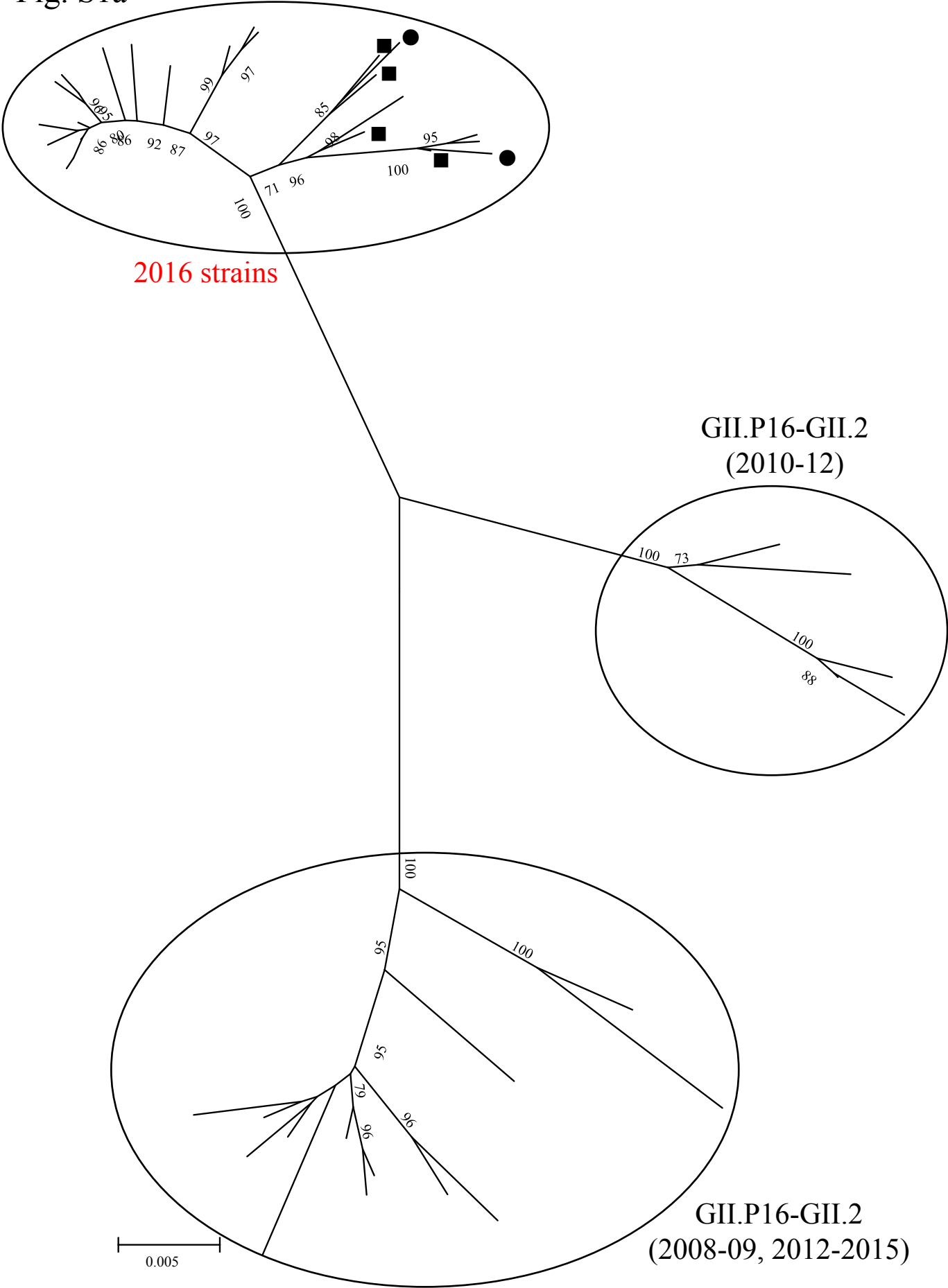

Fig. S1b

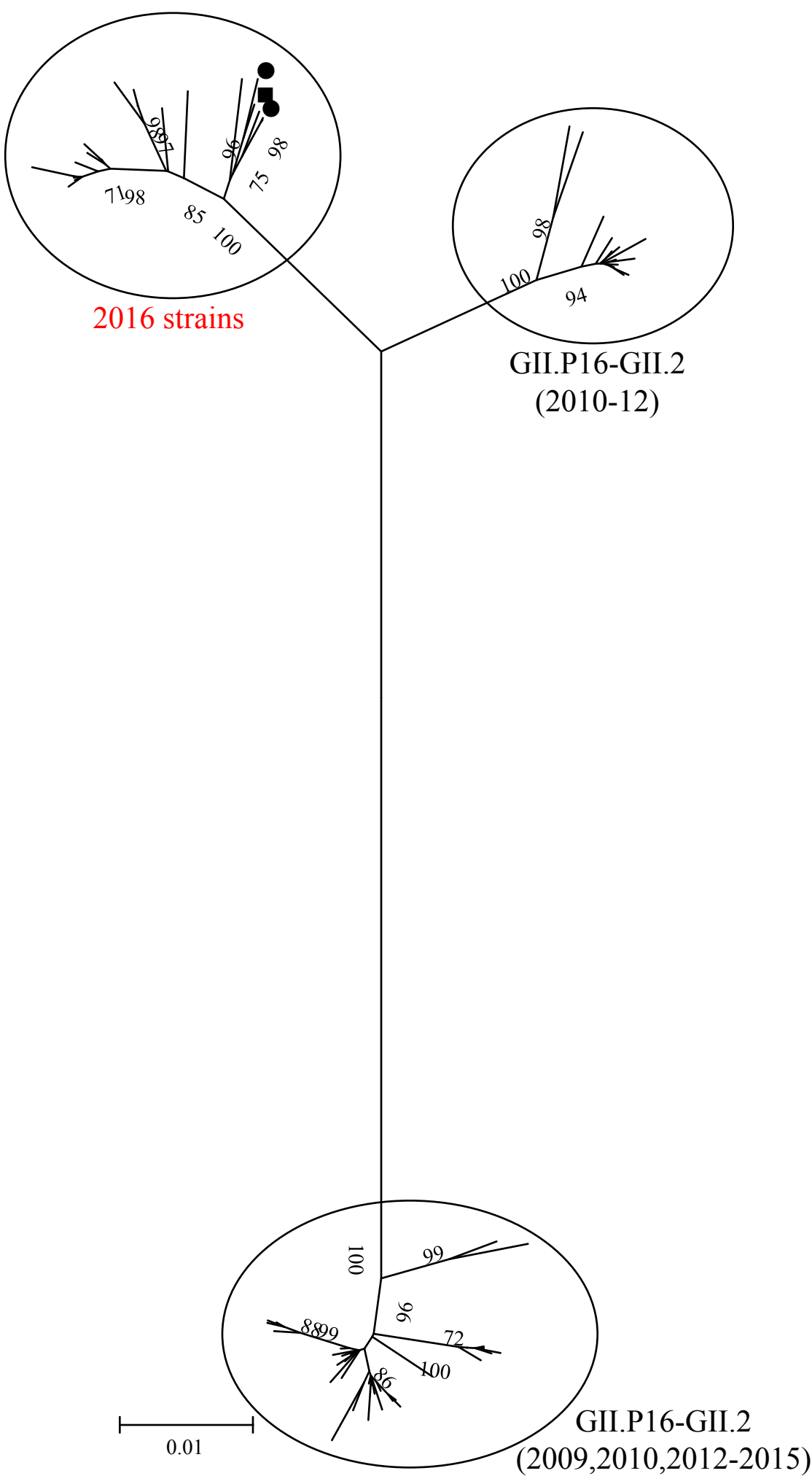

Supplement: FIGURE S1 — Phylogenetic trees for (a) the VP1 gene and (b) RdRp region in the HuNoV GII.P16-GII.2 strains were constructed using the neighbor-joining method. The scale bars represent substitution per site. Bootstrap values of more than 70% were shown. Circles indicate strains detected in the United States in the 2016/2017 winter season, and the square indicates the strain detected in China in the 2016/2017 winter season. [file Presentation_1.PDF]
