# Supplementary material for: Genetic Analysis of Human Norovirus Strains in Japan in 2016–2017
Source: Front Microbiol. 2018 Jan 18;9:1. doi: 10.3389/fmicb.2018.00001 (PMC5778136; doi:10.3389/fmicb.2018.00001)
Supplement: TABLE S1 — Primers used in this study. [file Table_1.DOCX]

| Table S1. Primers used in this study | | |
| --- | --- | --- |
| Coding region | Primer | Sequences |
| *RdRp* | GII.P16-PCR-1F | CCCAAARCCAATYAGACCAGATGT |
|  | GII.P16-Seq-1F | AGAATGGGRACACACGCAA |
|  | GII.P16-Seq-2F | AYGAGCCTGCCTACCTYGG |
|  | GII.P16-Seq-3F | HCTGCTYTGGGGCTCTGA |
|  | GII.P16-Seq-4F | AAGTCACCAAHCTGTCYCCTGACAT |
|  | GII.P16-Seq-5F | ACTYAAAGAAGGTGGGATGGACT |
|  | G2-SKR [1] | CCRCCNGCATRHCCRTTRTACAT |
| *VP1* | GII.2-PCR-Seq-1F | AYYTGAGCACGTGGGAGG |
|  | G2-SKF [1] | CNTGGGAGGGCGATCGCAA |
|  | G2-SKR [1] | CCRCCNGCATRHCCRTTRTACAT |
|  | GII.2-Seq-2F | YAAGTTRGTCTTCGCCGC |
|  | GII.2-Seq-3F | CCAGTGTCYATAGAYCAGATGTAC |
|  | GII.2-Seq-4R | CAGAAGGGGCRAGRTTTGT |
|  | GII.2-PCR-Seq-5R | GAACYRAGCCCATTGCTGA |

Reference

1. Kojima S, Kageyama T, Fukushi S, Hoshino FB, Shinohara M, Uchida K, et al. Genogroup-specific PCR primers for detection of Norwalk-like viruses. J Virol Methods. 2002 Feb;100(1-2):107-14.
