# Supplementary material for: Genetic Analysis of Human Norovirus Strains in Japan in 2016–2017
Source: Front Microbiol. 2018 Jan 18;9:1. doi: 10.3389/fmicb.2018.00001 (PMC5778136; doi:10.3389/fmicb.2018.00001)
Supplement: TABLE S2 — Strains used in this study. [file Table_2.DOCX]

Table S2. Strains used in this study.

| Strain names | Accession Number | Coding region (s) |
| --- | --- | --- |
| Hu/GII/JP/2011/GII.P16-GII.2/Saitama51 | LC209447 | RdRp, VP1 |
| Hu/GII/JP/2011/GII.P16-GII.2/Osaka9 | LC209448 | RdRp, VP1 |
| Hu/GII/JP/2010/GII.P16-GII.2/Kanagawa50 | LC209460 | RdRp, VP1 |
| Hu/GII/JP/2012/GII.P16-GII.2/Miyagi1 | LC145787 | RdRp, VP1 |
| Hu/GII/JP/2011/GII.P16-GII.2/Yamaguchi4 | LC209468 | RdRp, VP1 |
| Hu/GII/JP/2011/GII.P16-GII.2/Osaka038 | LC209452 | RdRp, VP1 |
| Hu/GII/JP/2011/GII.P16-GII.2/Osaka023 | LC209453 | RdRp, VP1 |
| Hu/GII/JP/2011/GII.P16-GII.2/Osaka18 | LC209451 | RdRp, VP1 |
| Hu/GII/JP/2011/GII.P16-GII.2/Ehime45 | LC209479 | RdRp, VP1 |
| Hu/GII/JP/2011/GII.P16-GII.2/Hiroshima26 | LC209471 | RdRp, VP1 |
| Hu/GII/JP/2010/GII.P16-GII.2/Kanagawa51 | LC209459 | RdRp, VP1 |
| Hu/GII/JP/2011/GII.P16-GII.2/Hokkaido17 | LC209467 | RdRp, VP1 |
| Hu/GII/JP/2012/GII.P16-GII.2/Ehime46 | LC209478 | RdRp, VP1 |
| Hu/GII/JP/2011/GII.P16-GII.2/Osaka26 | LC209449 | RdRp, VP1 |
| Hu/GII/JP/2012/GII.P16-GII.2/Saitama121 | LC209446 | RdRp, VP1 |
| Hu/GII/JP/2011/GII.2/Tokyo/10-4320 | AB629946 | VP1 |
| Hu/GII/JP/2010/GII.P16-GII.2/Ehime44 | LC209480 | RdRp, VP1 |
| Hu/GII/JP/2010/GII.P16-GII.2/Ehime43 | LC209481 | RdRp, VP1 |
| Hu/GII/US/2011/GII.P16-GII.2/HS255 | KJ407074 | RdRp, VP1 |
| Hu/GII/JP/2014/GII.P2-GII.2/Yamaguchi014 | LC209469 | RdRp, VP1 |
| Hu/GII/JP/2004/GII.P2-GII.2/Tochigi87 | LC209437 | RdRp, VP1 |
| Hu/GII/JP/2004/GII.P2-GII.2/Tochigi86 | LC209438 | RdRp, VP1 |
| Hu/GII/JP/2004/GII.P2-GII.2/Tochigi85 | LC209436 | RdRp, VP1 |
| Hu/GII/JP/2004/GII.P2-GII.2/Hokkaido13 | LC209464 | RdRp, VP1 |
| Hu/GII/JP/2004/GII.P2-GII.2/MK04 | DQ456824 | RdRp, VP1 |
| Hu/GII/JP/2004/GII.2/OC04071 | AB279564 | VP1 |
| Hu/GII/JP/2005/GII.2/OC05143 | AB662852 | VP1 |
| Hu/GII/JP/2006/GII.2/OC06005 | AB662854 | VP1 |
| Hu/GII/JP/2005/GII.2/OC05145 | AB662853 | VP1 |
| Hu/GII/JP/2004/GII.2/OC04042 | AB279558 | VP1 |
| Hu/GII/JP/2004/GII.2/OCS030697 | AB279571 | VP1 |
| Hu/GII/JP/2004/GII.2/OCS040035 | AB279572 | VP1 |
| Hu/GII/JP/2004/GII.2/OC04075 | AB279566 | VP1 |
| Hu/GII/JP/2004/GII.2/OC04056-1 | AB279560 | VP1 |
| Hu/GII/JP/2004/GII.2/OC04056-2 | AB279561 | VP1 |

Table S2. Strains used in this study (Continued).

| Strain names | Accession Number | Coding region (s) |
| --- | --- | --- |
| Hu/GII/JP/2004/GII.2/OC04073 | AB279565 | VP1 |
| Hu/GII/JP/2004/GII.2/OC04067 | AB279563 | VP1 |
| Hu/GII/JP/2004/GII.2/OC04038 | AB279557 | VP1 |
| Hu/GII/JP/2004/GII.2/OC04076 | AB279567 | VP1 |
| Hu/GII/JP/2004/GII.2/OC04059 | AB279562 | VP1 |
| Hu/GII/JP/2004/GII.2/OCS040100 | AB279573 | VP1 |
| Hu/GII/JP/2002/GII.2/OC02012 | AB279555 | VP1 |
| Hu/GII/NL/2002/GII.2/Heerlen7E | AB281086 | VP1 |
| Hu/GII/NL/2002/GII.2/Rotterdam39E | AB281087 | VP1 |
| Hu/GII/NL/2001/GII.2/Zwolle25E | AB281085 | VP1 |
| Hu/GII/NL/2003/GII.2/Leeuwarden71 | AB281088 | VP1 |
| Hu/GII/JP/2006/GII.P2-GII.2/Hokkaido14 | LC209462 | RdRp, VP1 |
| Hu/GII/NL/2005/GII.2/Vaals87 | AB281090 | VP1 |
| Hu/GII/NL/2005/GII.2/Goes28 | AB281089 | VP1 |
| Hu/GII/JP/2006/GII.2/OH06023 | AB662863 | VP1 |
| Hu/GII/JP/2008/GII.2/OH08019 | AB662867 | VP1 |
| Hu/GII/JP/2008/GII.2/OC08079 | AB662859 | VP1 |
| Hu/GII/NL/2001/GII.2/Leeuwarden15 | AB281084 | VP1 |
| Hu/GII/JP/1997/GII.2/OC97049 | AB279553 | VP1 |
| Hu/GII/JP/2000/GII.2/Maizuru/2840 | EF547398 | VP1 |
| Hu/GII/JP/2015/GII.P2-GII.2/Miyagi63 | LC209457 | RdRp, VP1 |
| Hu/GII/JP/2010/GII.P2-GII.2/Hokkaido16 | LC209465 | RdRp, VP1 |
| Hu/GII/JP/2010/GII.P2-GII.2/Hiroshima19 | LC209473 | RdRp, VP1 |
| Hu/GII/JP/2010/GII.P2-GII.2/Hiroshima18 | LC209472 | RdRp, VP1 |
| Hu/GII/JP/2010/GII.P2-GII.2/Hiroshima17 | LC209474 | RdRp, VP1 |
| Hu/GII/JP/2010/GII.2/Tokyo/10-14 | AB629941 | VP1 |
| Hu/GII/JP/2010/GII.2/OC10009 | AB662875 | VP1 |
| Hu/GII/JP/2009/GII.2/OC09103 | AB662872 | VP1 |
| Hu/GII/JP/2010/GII.2/OH10024 | AB662898 | VP1 |
| Hu/GII/JP/2009/GII.2/OC09104 | AB662873 | VP1 |
| Hu/GII/JP/2009/GII.2/OH09035 | AB662886 | VP1 |
| Hu/GII/JP/2010/GII.2/OH10008 | AB662891 | VP1 |
| Hu/GII/JP/2010/GII.2/OH10005-2 | AB662888 | VP1 |
| Hu/GII/JP/2010/GII.2/OH10029 | AB662901 | VP1 |
| Hu/GII/JP/2010/GII.2/OH10011-2 | AB662892 | VP1 |

Table S2. Strains used in this study (Continued).

| Strain names | Accession Number | Coding region (s) |
| --- | --- | --- |
| Hu/GII/JP/2010/GII.2/OC10026 | AB662879 | VP1 |
| Hu/GII/JP/2009/GII.2/OH09034 | AB662885 | VP1 |
| Hu/GII/JP/2008/GII.2/OH08029-2 | AB662869 | VP1 |
| Hu/GII/JP/2008/GII.2/OH08009 | AB662866 | VP1 |
| Hu/GII/JP/2008/GII.2/OC08124 | AB662860 | VP1 |
| Hu/GII/JP/2008/GII.2/OC080306 | AB535749 | VP1 |
| Hu/GII/JP/2007/GII.2/OH07013 | AB662865 | VP1 |
| Hu/GII/JP/2007/GII.2/OC07107 | AB662858 | VP1 |
| Hu/GII/JP/2007/GII.2/OH07001 | AB662864 | VP1 |
| Hu/GII/JP/2009/GII.2/OC09044 | AB662862 | VP1 |
| Hu/GII/JP/2009/GII.2/OC09094 | AB662871 | VP1 |
| Hu/GII/JP/2009/GII.2/OH09030 | AB662883 | VP1 |
| Hu/GII/JP/2008/GII.P2-GII.2/Hokkaido15 | LC209463 | RdRp, VP1 |
| Hu/GII/JP/2004/GII.P12-GII.2/Tochigi92 | LC209435 | RdRp, VP1 |
| Hu/GII/JP/2005/GII.2/OC05041 | AB662850 | VP1 |
| Hu/GII/JP/2006/GII.2/OC06014 | AB662856 | VP1 |
| Hu/GII/JP/2004/GII.2/OC04169 | AB279568 | VP1 |
| Hu/GII/JP/2002/GII.2/Ina/02 | AB195225 | VP1 |
| Hu/GII/JP/2015/GII.P2-GII.2/Saitama169 | LC209440 | RdRp, VP1 |
| Hu/GII/TW/2015/GII.2/15-DS-4 | KT962983 | VP1 |
| Hu/GII/JP/2013/GII.P16-GII.2/Tochigi46 | LC209431 | RdRp, VP1 |
| Hu/GII/JP/2012/GII.P16-GII.2/Hokkaido18 | LC209466 | RdRp, VP1 |
| Hu/GII/JP/2014/GII.P16-GII.2/Aomori7 | LC145798 | RdRp, VP1 |
| Hu/GII/JP/2014/GII.P16-GII.2/Akita7 | LC145799 | RdRp, VP1 |
| Hu/GII/JP/2014/GII.P16-GII.2/Akita8 | LC145800 | RdRp, VP1 |
| Hu/GII/JP/2013/GII.P16-GII.2/Miyagi8 | LC209455 | RdRp, VP1 |
| Hu/GII/JP/2014/GII.P16-GII.2/Miyagi2 | LC145801 | RdRp, VP1 |
| Hu/GII/JP/2014/GII.P16-GII.2/Kanagawa52 | LC209458 | RdRp, VP1 |
| Hu/GII/JP/2014/GII.P16-GII.2/Osaka4 | LC145802 | RdRp, VP1 |
| Hu/GII/JP/2014/GII.P16-GII.2/Saitama126 | LC209441 | RdRp, VP1 |
| Hu/GII/JP/2013/GII.P16-GII.2/Ehime9 | LC209475 | RdRp, VP1 |
| Hu/GII/JP/2013/GII.P16-GII.2/Ehime6 | LC209477 | RdRp, VP1 |
| Hu/GII/JP/2013/GII.P16-GII.2/Ehime8 | LC209476 | RdRp, VP1 |
| Hu/GII/JP/2014/GII.P16-GII.2/Ehime4 | LC145807 | RdRp, VP1 |
| Hu/GII/JP/2014/GII.P16-GII.2/Ehime5 | LC145808 | RdRp, VP1 |

Table S2. Strains used in this study (Continued).

| Strain names | Accession Number | Coding region (s) |
| --- | --- | --- |
| Hu/GII/JP/2012/GII.P16-GII.2/Tochigi30 | LC209432 | RdRp, VP1 |
| Hu/GII/JP/2013/GII.P16-GII.2/Saitama123 | LC209444 | RdRp, VP1 |
| Hu/GII/JP/2012/GII.P16-GII.2/Fukui2 | LC145790 | RdRp, VP1 |
| Hu/GII/JP/2012/GII.P16-GII.2/Saitama4 | LC145791 | RdRp, VP1 |
| Hu/GII/JP/2012/GII.P16-GII.2/Ehime2 | LC145797 | RdRp, VP1 |
| Hu/GII/JP/2014/GII.P16-GII.2/Tochigi17 | LC209434 | RdRp, VP1 |
| Hu/GII/JP/2012/GII.P16-GII.2/Saitama5 | LC145792 | RdRp, VP1 |
| Hu/GII/JP/2012/GII.P16-GII.2/Fukui1 | LC145789 | RdRp, VP1 |
| Hu/GII/JP/2013/GII.P16-GII.2/Saitama124 | LC209443 | RdRp, VP1 |
| Hu/GII/JP/2012/GII.P16-GII.2/Osakacity5 | LC145793 | RdRp, VP1 |
| Hu/GII/JP/2012/GII.P16-GII.2/Saitama122 | LC209445 | RdRp, VP1 |
| Hu/GII/JP/2012/GII.P16-GII.2/Tochigi26 | LC209433 | RdRp, VP1 |
| Hu/GII/JP/2014/GII.P16-GII.2/Hiroshima30 | LC209470 | RdRp, VP1 |
| Hu/GII/JP/2012/GII.P16-GII.2/Niigata5 | LC145788 | RdRp, VP1 |
| Hu/GII/JP/2012/GII.P16-GII.2/Ehime1 | LC145796 | RdRp, VP1 |
| Hu/GII/JP/2012/GII.P16-GII.2/Hiroshimacity2 | LC145794 | RdRp, VP1 |
| Hu/GII/JP/2014/GII.P16-GII.2/Hiroshimacity6 | LC145806 | RdRp, VP1 |
| Hu/GII/JP/2014/GII.2/Hiroshima6 | LC145804 | VP1 |
| Hu/GII/JP/2014/GII.P16-GII.2/Hiroshimacity5 | LC145805 | RdRp, VP1 |
| Hu/GII/JP/2012/GII.P16-GII.2/Hiroshimacity1 | LC145795 | RdRp, VP1 |
| Hu/GII/JP/2013/GII.P16-GII.2/Miyagi7 | LC209456 | RdRp, VP1 |
| Hu/GII/JP/2012/GII.P16-GII.2/Akita8 | LC145786 | RdRp, VP1 |
| Hu/GII/TW/2011/GII.2/CGMH47 | KC464505 | VP1 |
| Hu/GII/JP/2013/GII.P16-GII.2/Saitama125 | LC209442 | RdRp, VP1 |
| Hu/GII/JP/2014/GII.P16-GII.2/Osaka246 | LC228948 | RdRp, VP1 |
| Hu/GII/JP/2014/GII.P16-GII.2/Osaka225 | LC209450 | RdRp, VP1 |
| Hu/GII/JP/2014/GII.P16-GII.2/Osaka5 | LC145803 | RdRp, VP1 |
| Hu/GII/JP/2010/GII.P16-GII.2/Osaka019 | LC209454 | RdRp, VP1 |
| Hu/GII/JP/2009/GII.P16-GII.2/Kanagawa49 | LC209461 | RdRp, VP1 |
| Hu/GII/JP/2009/GII.2/OC09072 | AB662870 | VP1 |
| Hu/GII/JP/2010/GII.2/OH10013 | AB662894 | VP1 |
| Hu/GII/JP/2010/GII.2/OH10006 | AB662889 | VP1 |
| Hu/GII/JP/2009/GII.2/OH09028 | AB662881 | VP1 |
| Hu/GII/JP/2009/GII.2/OH09032 | AB662884 | VP1 |
| Hu/GII/JP/2010/GII.2/OH10015-2 | AB662895 | VP1 |

Table S2. Strains used in this study (Continued).

| Strain names | Accession Number | Coding region (s) |
| --- | --- | --- |
| Hu/GII/JP/2010/GII.2/OH10026 | AB662900 | VP1 |
| Hu/GII/JP/2010/GII.2/OH10025 | AB662899 | VP1 |
| Hu/GII/JP/2010/GII.2/OH10012 | AB662893 | VP1 |
| Hu/GII/JP/2010/GII.2/OH10020 | AB662896 | VP1 |
| Hu/GII/JP/2010/GII.2/OC10012-2 | AB662876 | VP1 |
| Hu/GII/JP/2010/GII.2/OC10058 | AB662880 | VP1 |
| Hu/GII/JP/2010/GII.2/OH10021 | AB662897 | VP1 |
| Hu/GII/JP/2008/GII.2/OH08020 | AB662868 | VP1 |
| Hu/GII/JP/2008/GII.2/OC08154 | AB662861 | VP1 |
| Hu/GII/NL/1999/GII.2/Coevorden191S | AB281081 | VP1 |
| Hu/GII/NL/2000/GII.2/DenHaag37 | AB281082 | VP1 |
| Hu/GII/NL/2000/GII.2/Delft48M | AB281083 | VP1 |
| Hu/GII/JP/2002/GII.2/OCS020289 | AB279570 | VP1 |
| Hu/GII/UK/1989/GII.2/Melksham | X81879 | VP1 |
| Hu/GII/US/1997/GII.2/Chesterfield/434 | AY054300 | VP1 |
| Hu/GII/MYS/1978/GII.P2-GII.2/KL109 | JX846925 | RdRp, VP1 |
| Hu/GII/1976/GII.P2-GII.2/Snow_Mountain | AY134748 | RdRp, VP1 |
| Hu/GII/JP/2014/GII.Pe-GII.2/Saitama127 | LC209439 | RdRp, VP1 |
| Hu/GII/JP/2005/GII.2/OC05010 | AB279569 | VP1 |
| Hu/GII/US/2002/GII.2/TCH-560 | KC998960 | VP1 |
| Hu/GII/US/2002/GII.2/NF2002 | JQ320072 | VP1 |
| Hu/GII/JP/2002/GII.2/OC02022 | AB279556 | VP1 |
| Hu/GII/JP/2004/GII.P22-GII.2/OsakaNI | DQ366347 | RdRp, VP1 |
| Hu/GII/JP/2001/GII.2/OC01243 | AB279554 | VP1 |
| Hu/GII/RUS/2011/GII.P16/Novosibirsk/Nsk-N1648 | KF944111 | RdRp |
| Hu/GII/RUS/2011/GII.P16/Novosibirsk/Nsk-N1659 | KF944110 | RdRp |
| Hu/GII/RUS/2012/GII.P16/Omsk/O1370 | KT779557 | RdRp |
| Hu/GII/RUS/2012/GII.P16/Smolensk/S12-31 | KF895841 | RdRp |
| Hu/GII/TW/2013/GII.P16/New/Taipei/13-BA-1 | KM036380 | RdRp |
| Hu/GII/JP/2016/GII.P16/Kawasaki194 | LC175468 | RdRp |
| Hu/GII/JP/2002/GII.P16/Saitama/T87 | KJ196286 | RdRp |
| Hu/GII/JP/2016/GII.P16-GII.2/Kawasaki129 | LC215413 | RdRp, VP1 |
| Hu/GII/JP/2016/GII.P16-GII.2/Kawasaki151 | LC215414 | RdRp, VP1 |
| Hu/GII/JP/2016/GII.P16-GII.2/Kawasaki121 | LC215415 | RdRp, VP1 |
| Hu/GII/JP/2015/GII.P16-GII.2/Ibaraki197 | LC213885 | RdRp, VP1 |

Table S2. Strains used in this study (Continued).

| Strain names | Accession Number | Coding region (s) |
| --- | --- | --- |
| Hu/GII/JP/2016/GII.P16-GII.2/Ibaraki253 | LC213886 | RdRp, VP1 |
| Hu/GII/JP/2016/GII.P16-GII.2/Ibaraki267 | LC213887 | RdRp, VP1 |
| Hu/GII/JP/2016/GII.P16-GII.2/Ibaraki273 | LC213888 | RdRp, VP1 |
| Hu/GII/JP/2016/GII.P16-GII.2/Ibaraki290 | LC213889 | RdRp, VP1 |
| Hu/GII/JP/2016/GII.P16-GII.2/Ibaraki324 | LC213890 | RdRp, VP1 |
| Hu/GII/JP/2016/GII.P16-GII.2/Ibaraki329 | LC213891 | RdRp, VP1 |
| Hu/GII/JP/2016/GII.P16-GII.2/Ibaraki374 | LC213892 | RdRp, VP1 |
| Hu/GII/JP/2016/GII.P16-GII.2/Ibaraki412 | LC213893 | RdRp, VP1 |
| Hu/GII/JP/2016/GII.P16-GII.2/Ibaraki423 | LC213894 | RdRp, VP1 |
| Hu/GII/JP/2016/GII.P16-GII.2/Ibaraki472 | LC213895 | RdRp, VP1 |
| Hu/GII/JP/2016/GII.P16-GII.2/Ibaraki518 | LC213896 | RdRp, VP1 |
| Hu/GII/JP/2016/GII.P16-GII.2/Ibaraki536 | LC213897 | RdRp, VP1 |
| Hu/GII/JP/2016/GII.P16-GII.2/Ibaraki602 | LC213898 | RdRp, VP1 |
| Hu/GII/JP/2016/GII.P16-GII.2/Ibaraki607 | LC213899 | RdRp, VP1 |
| Hu/GII/JP/2016/GII.P16-GII.2/Ibaraki636 | LC213900 | RdRp, VP1 |
| Hu/GII/JP/2016/GII.P16-GII.2/Ibaraki658 | LC213901 | RdRp, VP1 |
