# Supplementary material for: Genetic Analysis of Human Norovirus Strains in Japan in 2016–2017
Source: Front Microbiol. 2018 Jan 18;9:1. doi: 10.3389/fmicb.2018.00001 (PMC5778136; doi:10.3389/fmicb.2018.00001)
Supplement: TABLE S3 — Conditions of Bayesian Markov chain Monte Carlo method for the phylogenic trees. [file Table_3.DOCX]

| Table S3. Conditions of Bayesian Markov chain Monte Carlo method for the phylogenic trees | | | | | | |
| --- | --- | --- | --- | --- | --- | --- |
| Gene | Number of strains | Substitution model | Clock model | Demographic model | Length of MCMC chain | Log parameter |
| *VP1* | 186 | TN93-Γ | Relaxed Clock Exponential | Coalescent Constant Population | 100,000,000 | 1,000 |
| *RdRp* | 107 | GTR-Γ | Strict clock | Coalescent Constant Population | 100,000,000 | 1,000 |
