# Supplementary material for: Genetic Analysis of Human Norovirus Strains in Japan in 2016–2017
Source: Front Microbiol. 2018 Jan 18;9:1. doi: 10.3389/fmicb.2018.00001 (PMC5778136; doi:10.3389/fmicb.2018.00001)
Supplement: TABLE S4 — Conditions of Bayesian skyline plot analyses. [file Table_4.DOCX]

| Table S4. Conditions of Bayesian skyline plot analyses | | | | | |
| --- | --- | --- | --- | --- | --- |
|  | Number of strains | Substitution model | Clock model | Length of MCMC chain | Log parameter |
| All GII.2 strains (VP1) | 186 | TN93-Γ | Strick Clock | 100,000,000 | 1,000 |
| GII.P16 strains (RdRp) | 88 | TN93-Γ | Relaxed Clock Exponential | 100,000,000 | 1,000 |
